# The role of serotonin circuit dysregulation in neurobehavioural symptoms of Alzheimer's disease

**DOI:** 10.1002/alz70855_097919

**Published:** 2025-12-23

**Authors:** Derya Sargin

**Affiliations:** ^1^ University of Calgary, Calgary, AB, Canada

## Abstract

**Background:**

The dorsal raphe nucleus (DRN) serotonin (5‐HT) neurons, which are critical for socioemotional regulation and cognitive function, are among the earliest to exhibit tau pathology in Alzheimer's disease (AD). 5‐HT neurons play a pivotal role in mood, social behavior and cognition. Here, we developed a mouse model to study how early tau pathology in DRN 5‐HT neurons contributes to the neuropsychiatric symptoms of AD.

**Method:**

We generated a cre‐dependent adeno‐associated virus (AAV) to express a phosphorylation‐prone form of human tau (hTauP301L) (controls; fluorescent reporter only). We infused the virus into the DRN of Pet1‐cre mice, resulting in its specific expression in 5‐HT neurons. Four weeks post‐ infusion, mice underwent a series of behavioral tasks to assess tau‐induced changes in socioemotional and cognitive behaviors. A separate group was included in RNA‐seq analysis to investigate the mechanisms underlying early tau‐induced pathological changes.

**Result:**

Early pathological tau expression in 5‐HT neurons resulted in increased anxiety‐like behavior, altered stress coping strategies, female sex‐specific abnormal social behavior and mild cognitive impairment. We additionally identified altered molecular markers that may underlie tau‐induced early behavioral changes.

**Conclusion:**

Our findings offer mechanistic insight into the emergence of neuropsychiatric symptoms early in AD progression, highlighting key targets for therapeutic intervention.

